# Putative candidate genes responsible for leaf rolling in rye (*Secale cereale* L.)

**DOI:** 10.1186/s12863-018-0665-0

**Published:** 2018-08-09

**Authors:** Beata Myśków, Magdalena Góralska, Natalia Lenarczyk, Ilona Czyczyło-Mysza, Stefan Stojałowski

**Affiliations:** 1 Department of Plant Genetics, Breeding and Biotechnology, West-Pomeranian University of Technology, Szczecin, ul. Słowackiego 17, 71-434 Szczecin, Poland; 2 The Franciszek Górski Institute of Plant Physiology, Polish Academy of Science, ul. Niezapominajek 21, 30-239 Cracow, Poland

**Keywords:** Bulliform cells, COV, FEI1 (LRR receptor-like serine/threonine-protein kinase), Jasmonate O-methyltransferase, QTL, Subtilisin-like protease, TLP (tubby-like proteins), Transcription factor bHLH (basic helix-loop-helix protein)

## Abstract

**Background:**

Rolling of leaves (RL) is a phenomenon commonly found in grasses. Morphology of the leaf is an important agronomic trait in field crops especially in rice; therefore, majority of the rice breeders are interested in RL. There are only few studies with respect to RL of wheat and barley; however, the information regarding the genetic base of RL with respect to the shape of leaf in rye is lacking. To the best of our knowledge, this is the first study on the localization of loci controlling RL on high density consensus genetic map of rye.

**Results:**

Genotypic analysis led to the identification of 43 quantitative trait loci (QTLs) for RL, grouped into 28 intervals, which confirms the multigenic base of the trait stated for wheat and rice. Four stable QTLs were located on chromosomes 3R, 5R, and 7R.

Co-localization of QTL for RL and for different morphological, biochemical and physiological traits may suggests pleiotropic effects of some QTLs. QTLs for RL were associated with QTLs for such morphological traits as: grain number and weight, spike number per plant, compactness of spike, and plant height. Two QTLs for RL were found to coincide with QTLs for drought tolerance (4R, 7R), two with QTLs for heading earliness (2R, 7R), one with α-amylase activity QTL (7R) and three for pre-harvest sprouting QTL (1R, 4R, 7R).

The set of molecular markers strongly linked to RL was selected, and the putative candidate genes controlling the process of RL were identified. Twelve QTLs are considered as linked to candidate genes on the base of DArT sequences alignment, which is a new information for rye.

**Conclusions:**

Our results expand the knowledge about the network of QTLs for different morphological, biochemical and physiological traits and can be a starting point to studies on particular genes controlling RL and other important agronomic traits (yield, earliness, pre-harvest sprouting, reaction to water deficit) and to appoint markers useful in marker assisted selection (MAS). A better knowledge of the rye genome and genes could both facilitate rye improvement itself and increase the efficiency of utilizing rye genes in wheat breeding.

**Electronic supplementary material:**

The online version of this article (10.1186/s12863-018-0665-0) contains supplementary material, which is available to authorized users.

## Background

Leaf rolling (RL) is a typical response of a plant during water deficit that is observed in various field crops such as rice, maize, wheat, and sorghum. It decreases transpiration by decreasing the effective leaf area, and thus is a potentially useful drought tolerance mechanism in dry areas [1]. Although RL is a phenomenon commonly found in grasses, it has attracted much attention from rice researchers and breeders [2]. Only a few studies reported on RL of wheat [3–5] and barley [6], but studies regarding RL of rye are lacking.

Flag RL function is an important drought tolerance mechanism, enabling the plant to conserve water by decreasing transpiration during water stress and reduce leaf temperature [4]. Some Mediterranean grasses decrease transpiration as much as 46 to 63% by RL. In many species, RL does not occur until the water content in the leaf decreases to lethal levels (Parker 1968, after [7]).

In rice, RL is classified as abaxial leaf roll (both sides of the leaf roll inward along the vein) and adaxial leaf roll (both sides of the leaf roll outward along the vein) according to the direction of RL [8]. Analysis of some of the leaf development mutants of *Arabidopsis thaliana* and maize has shown that some mutations with respect to RL are related to the development of the leaf along the adaxial–abaxial axis. Establishment of leaf polarity and cell differentiation affecting leaf shape are controlled by both transcription factors and small RNAs [2, 9]. In higher plants, two types of cells are involved in RL: bulliform and hypodermis cells. Bulliform cells, which are located in the upper epidermis of the leaf near the midrib or vascular bundles of leaves, cause rolling in some *Gramineae* species such as rice, maize, wheat, and *Sorghum* spp. [1].

Due to its importance, many studies have been performed to characterize the genes controlling RL in rice. To date, no less than 17 rice mutants with rolled leaves have been characterized [10], no fewer than 70 genes/QTLs for the rolled leaf trait have been mapped or cloned throughout the rice genome [11], and at least 28 differentially expressed proteins related to RL traits have been isolated and identified [12]. A study on durum × wild emmer wheat recombinant inbred line population has reported 11 significant QTLs associated with flag RL mechanism [4]. In this study, we aimed to localize the loci controlling RL on high density consensus genetic map of rye. We also aimed to detect QTL co-localization of RL and other agronomic traits.

## Methods

### Plant material and genetic map

In this study, we used a population of recombinant inbred lines (RILs) namely, RIL-M, which is a cross between S120 and S76 lines. S120 and S76 were developed within the commercial breeding programs conducted at Danko Plant Breeding Ltd. (Choryń, Poland) and are partially related but are genetically different [13] with respect to the following: α-amylase activity (AA), preharvest sprouting (PHS), heading and flowering time (HE), and different morphological traits.

Mapping population consisted of 143 genotypes of RIL-F_8_ generation. Consensus genetic map for the RIL-M (with consideration of the data from four rye populations) was created using the Multipoint Consensus 2.2 software package [14]. Detailed information on RIL-M mapping population and algorithms used to release genetic map are provided by Milczarski et al. [15].

All seven linkage groups accounted for 1318 markers (1256 DArTs, i.e. markers detected by Diversity Array Technology and 62 PCR-based loci). Individual chromosomes included from 117 (5R) to 257 (6R) loci and spanned the distance of 128 cM (5R)–251 cM (7R). The whole length of map was 1355 cM, and average distance varied from 0.7 cM to 1.9 cM.

### Phenotype analyses

All the experiments in this study were conducted at West Pomeranian University of Technology, Szczecin (53.45°N, 14.53°E). The RIL-M population (S7–S12 generations) consisted of 143 genotypes and were analyzed during six vegetation seasons (years: 2008, 2009, 2010, 2012, 2013, and 2017). In the years 2008–2010, genotypes of mapping population were planted and analyzed in duplicates. Each RIL was represented by 7–8 plants grown in the field or in pots under natural or near natural conditions.

Due to inbreeding depression of numerous lines, individuals representing each genotype were first germinated in a glasshouse (15th–25th September) and then (1st–20th October) vital seedlings were planted manually in the field—each line into one row. Finally, 7–8 individuals were grown in each row (length of rows was 100 cm, dimension between rows was 17.5 cm). The order of lines grown in the field was random and different in each year of study for the following replicates: RL08–1, RL09–1, RL09–2, RL10–1, RL10–2, RL12, and RL17. Variants RL09–1 and RL09–2 as well as RL10–1 and RL10–2 were grown in different plots.

Genotypes of two variants (replicates RL08–2 and RL13) were grown in the buckets of 10 dm^3^ volume filled with an equal mass (1440 g) of soil and sand mixture (1:1 *v*/v), seven plants of each genotype together in one bucket. Seeds were sown in January; initially plants were grown at a temperature of 15 °C for first 3 weeks, followed by gradually decreasing the temperature until it approached atmospheric conditions. Further vegetation proceeded under natural conditions of the winter–summer period (February–August), with natural daylight duration.

**Fig. 1:**
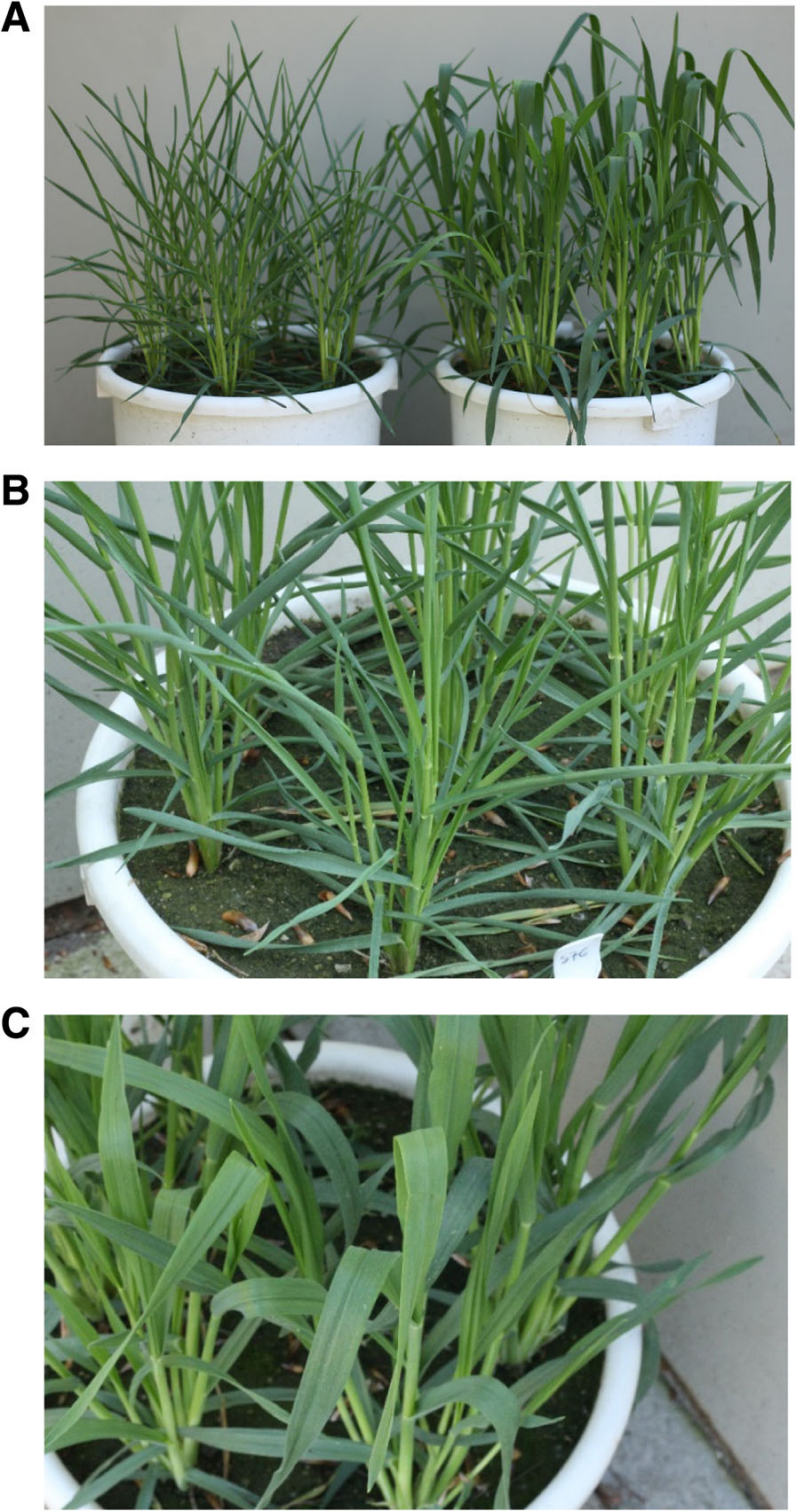
Parental inbred lines of rye mapping population RIL-M, differing in terms of rolling leaves. **a** Paternal line S76 with rolled leaves, maternal line S120 with straight leaves, (**b**) line S76, and (**c**) line S120

Our plant material was characterized by adaxial rolling leaves. A visual score of the degree of RL was made on the whole plants at the tillering stage, using a 5-point scale: 1—no signs of rolling, 1.5—the first evidence of rolling, 2—slightly rolled leaves, 2.5—strongly rolled leaves, and 3—completely rolled leaves (a closed cylinder). Figure 1 shows the parental lines with extreme rate of traits. The assessment was performed after rainfall or watering the plants in buckets, to avoid the effect of leaves rolling under the influence of water deficit.

### Statistical analysis

QTL mapping was performed using the method of composite interval mapping (CIM) with Windows QTL Cartographer 2.5 software [8]. The step size chosen for all QTLs was 2 cM. Significant thresholds to declare the presence of a QTL were estimated from 1000 permutations of the data at *P* < 0.05. In addition, nonparametric Fisher’s test was conducted to point markers significantly connected with rolling leaves segregations.

Variation parameters and correlation coefficient between replicates was established with STATISTICA 12.0 software package (Stat-Soft, Inc., USA, http://www.statsoft.com).

Results of CIM were compared with previous QTL analyses for AA, PHS, HE [16], with recalculated data for consensus map), morphological traits [17], spike compactness, and drought index of morphological traits [18] for the same genetic map of RIL-M population.

## Results

### Phenotypic variation and correlation analyses

The mapping population RIL-M was characterized by an average coefficient of variation of 32.15–44.51 in terms of RL, depending on the replication (Table 1). Mean values of the trait ranged from 1.74 to 2.11.

**Table 1:** Parameters of phenotypic variation and correlation coefficient (r) of leaf rolling (RL) in rye mapping population RIL-M, assessed in nine experimental replications

Experiment replication	Number of genotypes evaluated N	RL parameter Av	Variance	SD	Coefficient of variation	SE	Skewness	Kurtosis	r	
									Min	Max
RL08–1	139	1.92	0.71	0.84	43.90	0.07	0.15	−1.58	0.36*	0.60*
RL08-2	90	1.76	0.59	0.77	43.80	0.08	0.45	−1.17	0.25	0.60*
RL09-1	142	1.83	0.61	0.78	42.64	0.07	0.31	−1.30	0.29*	0.47*
RL09-2	140	1.74	0.51	0.71	40.93	0.06	0.42	−0.95	0.19	0.40*
RL10-1	138	1.82	0.60	0.78	42.66	0.07	0.33	−1.27	0.26	0.60*
RL10-2	131	1.75	0.61	0.78	44.51	0.07	0.47	−1.20	0.25	0.48*
RL12	128	1.94	0.51	0.71	36.78	0.06	0.19	−1.14	0.38*	0.56*
RL13	127	2.08	0.52	0.72	34.59	0.06	−0.12	−1.17	0.29	0.46*
RL17	135	2.11	0.46	0.68	32.15	0.06	−0.17	−1.12	0.29*	0.60*

*significant at *P* = 0.01

Significant positive correlations were found between results of the most pairs of replicates, and there was no correlation found in four cases (Additional file 1). Assessment of two variants in the same vegetative season showed a correlation coefficient of 0.60, 0.35, and 0.48 in the year 2008, 2009, and 2010, respectively. The weakest correlation (0.19) was noted between RL08–1 and RL09–2 and the strongest (0.60) correlation was found between RL08–2 and three other replications: RL08–1, RL10, RL17.

### QTL analyses

**Fig. 2:**
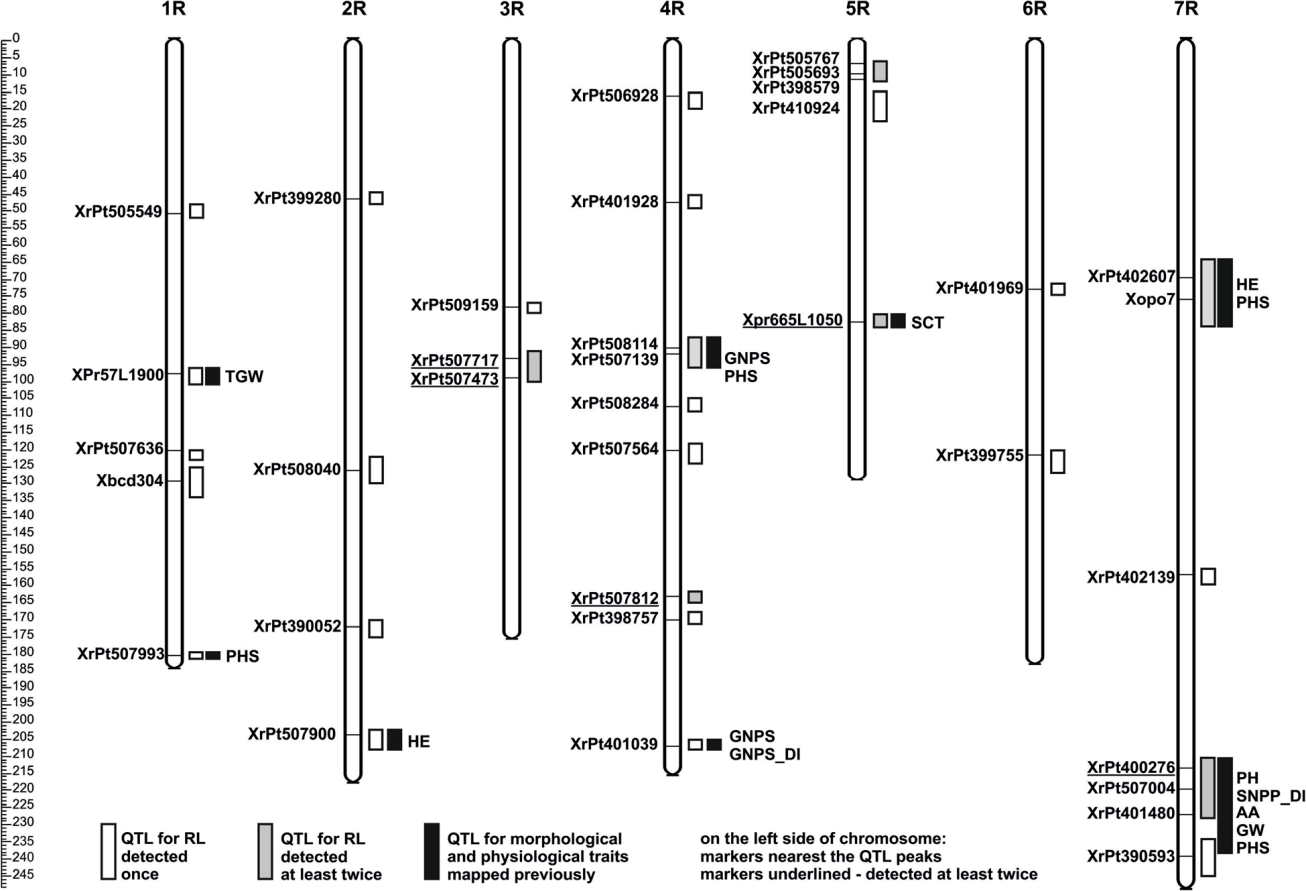
QTLs for leaf rolling (RL) detected on genetic map of rye population RIL-M. On the left side of chromosomes—markers nearest to the QTL peak, underlined—markers pointed as the nearest the QTL peak at least twice. On the right side of chromosomes—white rectangle—QTL detected once out of nine replicates, gray rectangle—QTL detected at least twice, black rectangle—QTL for other traits mapped previously in this population. Abbreviations: QTLs, quantitative trait loci; TGW, thousand grain weight; PHS, preharvest sprouting; HE – heading earliness; GNPS, grain number *per* spike; GNPS_DI, grain number *per* spike drought index; SCT, spike compactness; SNPP_DI, spike number per plant drought index; PH, plant height; AA, α-amylase activity; GW, grain weight per spike

Putative QTLs for RL were detected in each season except the year 2013. A total of 43 QTLs grouped in 28 intervals on all 7 chromosomes (2–8 *per* chromosome) were identified (Fig. 2). There were seven groups of coinciding QTLs found on chromosomes 3R, 4R, 5R, and 7R; four of them were detected 3–5 times (Fig. 2, Table 2).

**Table 2:** QTLs for leaf rolling (RL) in rye mapping population RIL-M, detected at least twice, out of nine replications

Experiment replication	QTL position			LOD	a	r^2^	Marker nearest to the QTL peak
	Chromosome	Peak [cM]	Interval [cM]				
RL10–1	3R	94	91–96	2.09	−0.3	6.73	XrPt507717
RL17	3R	95	92–98	3.55	−0.24	11.4	XrPt507717
RL12	3R	98	94–101	2.25	−0.22	8.17	XrPt507473
RL08–1	3R	99	96–100	4.48	−0.35	12.12	XrPt507473
RL08–2	3R	99	95–101	3.12	−0.31	13	XrPt507473
RL09–1	4R	90	87–96	3.21	−0.51	11.73	XrPt508114
RL17	4R	91	91–92	3.61	−0.33	11.93	XrPt507139
RL08–2	4R	164	162–164	3.58	−0.51	15.39	XrPt507812
RL09–2	4R	164	162–165	2.59	−0.38	7.75	XrPt507812
RL17	5R	6	5–9	4.51	−0.24	11.91	XrPt505767
RL08–1	5R	9	8–12	7.34	−0.4	21	XrPt505693
RL10–1	5R	12	7–12	2.73	−0.23	8.09	XrPt398579
RL09–1	5R	83	81–84	5.38	−0.35	18.59	Xpr665L1050
RL10–1	5R	83	80–84	2.94	−0.29	10.06	Xpr665L1050
RL08–1	5R	84	82–84	4.01	−0.31	9.85	Xpr665L1050
RL12	7R	69	64–76	2.58	−0.21	7.95	XrPt402607
RL17	7R	76	74–84	3.46	−0.32	9.17	Xopo7
RL09–2	7R	213	212–215	3.67	−0.26	9.41	XrPt400276
RL17	7R	215	210–216	3.39	−0.25	10.18	XrPt400276
RL12	7R	221	210–221	4.24	−0.27	13.52	XrPt507004
RL08–2	7R	225	215–226	3.64	−0.35	17.84	XrPt505931

a – additive effect of the maternal allele, r^2^ – variance explained by a QTL

The QTLs detected were found to be responsible for 6.61–21.00% of the variation in the trait. Eighteen loci were characterized with coefficient of variation more than 10%. LOD values ranged from 2.09 to 7.34. Twenty-two QTLs peaks achieved LOD value above 3.0. The highest LOD was achieved by locus from 5RS. Absolute value of parameter *a* ranged from 0.21 to 0.51. Most of the alleles causing RL (31; 16 if consider common localization) were contributed by S76; however, 12 alleles responsible for RL originated from S120.

Thirty-five markers were indicated as the nearest to the QTL peaks: 31 DArTs, 2 random amplified polymorphic DNAs (RAPDs), and 2 sequence characterized amplified regions (SCARs); among them, three loci were revealed twice (XrPt507717, XrPt507812, and XrPt400276), and two loci were revealed three times (XrPt507473 and Xpr665L1050).

Figure 2 shows the location of all QTLs. If any locus was found in different replicates, then we presented those only as a single gray rectangle.

In addition to CIM, the nonparametric, Fisher’s (*F*) test was used to detect markers significantly linked to the phenotype of rolled leaves. A total of 155 loci from 6 chromosomes were pointed as linked to the trait at least once, most of them (46) from chromosome 7R. Chromosome 1R had no representation; 2R was represented by one marker. By limiting the pool of markers to those detected at least thrice, a set of 128 loci was obtained (Table 3). Only 15 out of 35 loci nearest to the QTL peaks were in this group. Two strongly linked markers from 7R were revealed by *F* test in all 9 replications of the experiment. The other 26 markers were detected 8 times; among them, 1 marker from 5R, 3 from 3R, and the rest from 7R (Table 3). Next, 26 loci were found to be significant in 7 replications, 19 markers were pointed 6 times, 40 markers were pointed 5 times, 7 markers were pointed 4 times, 8 markers were pointed 3 times, and 10 markers were pointed twice. All intervals containing markers detected in *F* test included QTL designated by CIM method.

**Table 3:** Markers linked to leaf rolling (RL), significant in Fisher’s test at *P* = 0.01, at least in three replicates

Marker	1	2	3	4	5	Marker	1	2	3	4	5	Marker	1	2	3	4	5
XrPt410763	7R	221	93	22	9	XrPt402377	5R	5	96	25	7	XrPt390585	5R	121	43	11	5
XrPt410830	7R	222	96	22	9	XrPt402405	7R	240	66	18	7	XrPt398800	5R	121	70	20	5
XrPt346184	7R	224	97	20	8	XrPt410818	3R	92	75	19	7	XrPt400297	5R	98	50	13	5
XrPt389258	7R	222	96	24	8	**XrPt505767**	5R	6	103	27	7	XrPt400848	3R	84	60	15	5
**XrPt390593**	7R	240	116	24	8	XrPt506147	3R	89	72	17	7	XrPt401228	3R	84	52	14	5
XrPt390750	7R	251	110	21	8	XrPt506260	7R	240	69	17	7	XrPt401454	5R	17	69	17	5
**XrPt398579**	5R	12	103	29	8	XrPt507180	5R	12	112	34	7	XrPt401481	3R	108	43	13	5
XrPt399775	7R	233	68	14	8	XrPt507500	5R	5	103	27	7	XrPt402245	5R	16	76	20	5
XrPt400252	7R	224	90	17	8	**XrPt507717**	3R	94	82	18	7	XrPt402531	5R	125	47	16	5
XrPt400319	3R	89	63	13	8	XrPt507948	5R	0	83	23	7	**XrPt410924**	5R	15	92	22	5
XrPt400793	7R	233	68	14	8	XrPt508061	7R	72	68	14	7	XrPt411109	5R	113	57	16	5
XrPt401200	7R	227	114	19	8	**Xpr665L1050**	5R	84	85	22	6	XrPt411252	5R	17	76	20	5
XrPt401372	7R	233	75	15	8	XrPt346908	3R	78	56	14	6	XrPt411320	5R	119	56	16	5
**XrPt401480**	7R	227	94	16	8	XrPt347574	7R	65	71	16	6	XrPt505219	7R	65	47	17	5
XrPt401828	7R	247	121	24	8	XrPt347998	5R	14	92	22	6	**XrPt505693**	5R	8	89	29	5
XrPt402262	7R	247	109	21	8	XrPt398502	3R	100	69	17	6	XrPt505721	5R	113	57	16	5
XrPt410884	7R	233	68	14	8	XrPt398519	7R	65	71	16	6	XrPt506001	5R	17	76	20	5
XrPt505215	3R	91	67	13	8	XrPt401081	3R	100	69	17	6	XrPt506821	5R	102	48	13	5
XrPt505523	7R	233	68	14	8	XrPt401795	7R	65	71	16	6	XrPt506905	3R	79	51	14	5
XrPt505864	7R	224	97	20	8	XrPt402589	3R	76	57	15	6	XrPt507369	3R	84	52	14	5
**XrPt505931**	7R	224	97	20	8	**XrPt402607**	7R	68	74	17	6	XrPt507926	5R	98	50	13	5
XrPt506494	7R	222	96	20	8	XrPt410783	5R	12	92	22	6	XrPt507953	5R	122	72	20	5
XrPt506607	3R	91	67	13	8	XrPt411522	5R	121	57	14	6	XrPt508905	5R	98	50	13	5
XrPt506764	7R	224	95	19	8	XrPt505593	3R	89	77	19	6	XrPt508925	5R	7	82	24	5
XrPt507754	7R	222	96	21	8	XrPt506874	3R	103	48	14	6	**XrPt509159**	3R	79	62	17	5
XrPt507936	7R	222	91	21	8	**XrPt507004**	7R	220	81	18	6	Xscm141	5R	79	53	12	5
XrPt508559	7R	72	86	16	8	XrPt507462	3R	76	57	15	6	Xscsz877L950	5R	121	59	16	5
XrPt508837	7R	222	96	20	8	**XrPt507473**	3R	99	95	25	6	XrPt346583	5R	108	36	11	4
**Xopo7**	7R	76	82	18	7	XrPt508197	5R	5	98	27	6	XrPt390741	7R	208	48	14	4
Xpr57L470	5R	101	68	15	7	XrPt509647	3R	78	56	14	6	XrPt399654	7R	209	44	12	4
XrPt117252	3R	89	72	17	7	Amy3.2	5R	128	50	13	5	XrPt411020	5R	121	55	15	4
XrPt119718	5R	84	51	11	7	Xpr665L430	3R	81	41	10	5	XrPt411184	5R	18	59	16	4
XrPt120990	3R	89	72	17	7	XrPt346755	5R	17	76	20	5	XrPt505437	7R	208	50	13	4
XrPt345439	3R	89	72	17	7	XrPt346892	5R	17	76	20	5	XrPt509051	5R	106	43	12	4
XrPt348093	3R	89	72	17	7	XrPt346946	5R	17	76	20	5	XrPt346779	6R	59	35	16	3
XrPt389261	5R	12	108	32	7	XrPt346980	5R	17	76	20	5	XrPt347114	6R	63	33	16	3
XrPt389585	7R	216	81	17	7	XrPt347072	5R	17	76	20	5	XrPt389711	7R	207	38	13	3
XrPt389895	5R	5	103	27	7	XrPt347212	5R	16	79	21	5	XrPt390442	6R	59	35	16	3
XrPt389959	7R	210	96	23	7	XrPt347454	5R	17	76	20	5	XrPt400509	7R	207	34	11	3
XrPt390362	5R	6	96	25	7	XrPt347809	5R	17	76	20	5	XrPt400732	7R	202	38	13	3
XrPt398627	3R	89	72	17	7	XrPt389454	3R	81	53	14	5	XrPt401523	7R	207	48	12	3
**XrPt400276**	7R	214	96	23	7	XrPt389759	5R	107	38	12	5	XrPt509176	7R	207	48	12	3
XrPt401754	7R	73	55	12	7	XrPt389815	3R	81	53	14	5						

Bolded—markers nearest to the QTL peak, underlined—markers with predicted function (Table 4), 1—chromosome, 2—position [cM], 3—sum of *F* statistic from all replicates, 4—maximal value of *F* statistic, 5—number of replicates, in which marker was pointed as significant

### Markers of RL putative homologs

The set of DArT sequences [19] was screened to find sequences of markers linked to RL. A total of 67 found sequences were directed to BLAST sequence analysis in NCBI nucleotide collection database (https://www.ncbi.nlm.nih.gov/), using megaBLAST algorithm. Hits exceeding a score of 200 or identity of 95% and those with known (predicted) identity were chosen. Finally, 12 records met the set criteria (Table 4); one of them was matched to *Secale cereale* cds, one to *Triticum aestivum* genomic sequence, and the rest to *Aegilops tauschii* mRNA.

**Table 4:** DArTs linked to leaf rolling (RL) with known sequences, annotated in NCBI

Marker			NCBI annotation							
Marker	DArT sequence length (bp)	Chrom.	Description	Species	Max score	Total score	Query cover	E value	Identity	Accession
XrPt506905	665	3R	subtilisin-like protease SBT2.2 (LOC109761160), mRNA	*Aegilops tauschii subsp. tauschii*	97	97	8%	6.00E-16	95%	XM_020319955.1
**XrPt507717**	678	3R	protein LIKE COV 2 (LOC109734806), mRNA	*Aegilops tauschii* subsp. *tauschii*	279	279	24%	5.00E-71	96%	XM_020293989.1
**XrPt507473**	599	3R	Fhb1 region genomic sequence	*Triticum aestivum cv Sumai 3*	60	60	5%	7.00E-05	97%	KX907434.1
XrPt401081 XrPt398502	545	3R	jasmonate O-methyltransferase-like (LOC109744228), mRNA	*Aegilops tauschii* subsp. *tauschii*	359	692	86%	5.00E-95	95%	XM_020303320.1
XrPt508197	720	5R	RGA1-G gene, complete cds	*Secale cereale*	220	358	19%	4.00E-53	96%	KT725818.1
XrPt410783	463	5R	cancer-related nucleoside-triphosphatase (LOC109750338), mRNA	*Aegilops tauschii* subsp. *tauschii*	527	624	75%	1.00E-145	94%	XM_020309304.1
XrPt401454	218	5R	LRR receptor-like serine/threonine-protein kinase FEI 1 (LOC109774869), mRNA	*Aegilops tauschii* subsp. *tauschii*	57	57	13%	3.00E-04	100%	XM_020333663.1
XrPt400297	773	5R	serine/threonine-protein kinase AFC1-like (LOC109779169), transcript variant X4, mRNA	*Aegilops tauschii* subsp. *tauschii*	228	282	19%	2.00E-55	100%	XM_020337784.1
XrPt402531	422	5R	transcription factor bHLH79-like (LOC109737374), mRNA	*Aegilops tauschii* subsp. *tauschii*	99	99	13%	1.00E-16	98%	XM_020296517.1
**XrPt402607**	551	7R	polyadenylate-binding protein-interacting protein 7-like (LOC109738116), transcript variant X4, mRNA	*Aegilops tauschii* subsp. *tauschii*	470	470	60%	2.00E-128	93%	XM_020297211.1
**XrPt401480**	561	7R	tubby-like F-box protein 12 (LOC109763585), transcript variant X3, mRNA	*Aegilops tauschii* subsp. *tauschii*	601	601	65%	8.00E-168	96%	XM_020322436.1
XrPt390593	451	7R	vegetative cell wall protein gp1-like (LOC109748681), transcript variant X4, mRNA	*Aegilops tauschii* subsp. *tauschii*	57	57	6%	6.00E-04	100%	XM_020307705.1

Bolded—markers nearest to the QTL peak

### Co-localization of QTLs

Some QTL intervals for RL overlapped partially or completely with QTLs for other agronomic traits analyzed previously in this population. There were nine QTLs overlapping most precisely, which means that they had the same markers nearest to the QTL peak and nearly the same intervals (Fig. 2, Table 5).

**Table 5:** QTLs for leaf rolling (RL) in rye mapping population RIL-M, coinciding with QTLs for other traits, mapped previously in this population

Chrom.	Coinciding QTLs	Marker nearest to the QTL peak	QTL peak [cM]	QTL interval [cM]	LOD	a	r^2^
1R	RL08–1	Xpr57L1900	98	96–101	2.7	0.33	8.61
	TGW09		98	98–102	3.03	−1.94	9.42
1R	RL12	XrPt507993	181	180–182	2.8	0.24	8.57
	PHS08		181	178–183	3.02	−6.05	8.37
2R	HE08	Xrpt507900	202	200–206	3.33	0.55	8.74
	RL08–1		204	202–208	3.1	−0.31	9.63
4R	GNPS08	XrPt508114	89	87–91	3.05	6.77	10.8
	PHS10		89	85–106	2.69	−8.64	9.2
	RL09–1		90	87–96	3.21	− 0.51	11.73
4R	GNPS08	XrPt401039	207	206–208	4.47	−3.94	12.54
	RL10–2		207	205–208	3.36	−0.36	10.77
	GNPS_DI		207	205–210	2.73	12.76	15.45
5R	SCT09	Xpr665L1050	81	73–84	2.8	−0.1	12.02
	RL09–1		81	81–84	5.38	−0.35	18.59
	RL10–1		83	80–84	2.94	−0.29	10.06
	RL-08-1		84	82–84	4.01	−0.31	9.85
7R	RL17	Xopo7	76	74–84	3.46	−0.32	9.17
	PHS07		77	69–87	2.74	13.76	18.49
	HE09		84	74–95	2.3	0.83	16.97
7R	SNPP_DI	XrPt507004	219	214–228	1.85	−8.38	9.9
	PH08		220	216–222	2.76	4.15	8.31
	RL12		221	210–221	4.24	−0.27	13.52
7R	AA09	XrPt401480	227	226–238	8.25	1.21	22.85
	RL10–1		227	227–228	3.68	−0.28	11.51
	PHS08N		228	227–238	5.17	9.89	16.64
	GW09		229	224–229	2.11	−0.09	6.62

a – additive effect of the maternal allele, r^2^ – variance explained by a QTL

*TGW* thousand grain weight, *PHS* preharvest sprouting, *HE* heading earliness, *GNPS* grain number *per* spike, *GNPS_DI* grain number *per* spike drought index, *SCT* spike compactness, *SNPP_DI* spike number *per* plant drought index, *PH* plant hight, *AA* α-amylase activity, *GW* grain weight *per* spike

QTLs for RL were associated with QTLs for morphological traits such as grain number and weight, spike number per plant, compactness of spike, and plant height. Two QTLs of this set were loci controlling drought index (GNPS_DI and SNPP_DI), that is, those that caused differences in trait expression in normal and drought conditions. QTL for RL were also co-localized with two QTLs for heading earliness (2R, 7R), one with α-amylase activity QTL (7R) and three for pre-harvest sprouting QTL (1R, 4R, and 7R).

Distal part of chromosome 7RL is a special region with numerous overlapping QTLs: five controlling RL and five engaged in the expression of other traits (Fig. 2, Table 5).

## Discussion

RL is a phenomenon commonly found in grasses and quite commonly described for field crops, but it has not been studied in rye. As leaf morphology is an important agronomic trait in the breeding of rice [20], the mechanism of RL was of interest, especially to rice researchers. There is no information about the genetic base of molecular mechanisms involved in leaf shape in rye, except one report regarding QTLs controlling leaf area [21].

A study similar to ours has been conducted on tetraploid wheat [4]; however, we did not study RL as the reaction to water deficit. The research on drought resistance in durum wheat × wild emmer wheat recombinant inbred line population allowed to detect 11 significant QTLs associated with flag RL, mapped on chromosomes: 1A, 2A, 2B, 4B, 5A, 5B, 6A, 6B, 7A, and 7B [4]. Three of these QTLs were found to be environment responsive.

We detected 43 QTLs, grouped into 28 intervals, which confirms the multigenic base of the trait stated for wheat and rice. Studies on rice showed that no fewer than 70 genes/QTLs for RL have been mapped or cloned till now [11]. Four QTLs for RL were stable in different environments. Due to many QTLs for RL were detected once, their genotype×environment interaction (GEI) could be inferred. GEI is a common characteristic for quantitative traits. For possible breeding purposes (like marker Assisted Selection - MAS), QTLs that are more environment-specific should be treated with caution and more attention should be paid to the repetitive QTLs. However, QTLs of varying manifestation, dependent on the environmental influence are also interesting for cognitive purposes.

In this study, we focused on the analysis of RL per se, without linking this trait with the response to drought. However, co-localization of two QTLs for RL with QTLs for drought index of grain number per spike (4R) and spike number per plant (7R) revealed in other experiments [18] indicates the relationship of detected loci with adaptive mechanisms to drought-related stress conditions.

Furthermore, 5 out of 11 QTLs for RL mapped on tetraploid wheat were co-localized with QTLs associated with plant productivity [4]. RL was also found to be associated with plant height in four regions (2B, 4B, 6A, and 7A) and with heading earliness (days from planting to heading) in two intervals on chromosomes 4B and 5A [4]. We also found nine QTLs co-localized with QTLs for other agronomic traits mapped in the same population [17], such as grain number and weight, spike number per plant, compactness of spike and plant height. QTLs for RL were also found to be co-localized with two QTLs for heading earliness (2R and 7R), one with α-amylase activity QTL (7R), and three with preharvest sprouting QTLs (1R, 4R, and 7R) [16].

Additional confirmation of the association between loci engaged in controlling RL and QTLs responsible for different agronomic traits is the result of comparing markers linked with RL and markers for nine features studied in the other, unrelated rye RILs’ mapping population 541 × Ot1–3 [21]. There were loci for plant height, stem thickness, spike length, awn length, heading date, thousand grain weight, grain length, leaf area, and chlorophyll content localized on the DArT-based high-density map of this population.

Each of the nine traits was characterized by some markers common with these, linked to RL in our population. There were 45 such markers distributed throughout the five chromosomes (3R, 4R, 5R, 6R, and 7R). Among them three DArTs from 7R were common for RL and leaf size (XrPt505931, XrPt389959, and XrPt400276). Seven DArTs were linked to RL and chlorophyll content: three from 5R (XrPt389759, XrPt346583, and XrPt505721) and four from 7R chromosome (XrPt402607, XrPt398519, XrPt347574, and XrPt401795). All these relationships suggest very strong linkages and/or pleiotropic effects of many genes, which remains in agreement with previous results for rye [16, 17, 21], wheat [4], and rice [22].

All DArTs significantly linked to RL were subjected to screening the DArT sequences database, followed by NCBI database blasting, in order to find the homologs. A total of 12 records with a known identity were found; majority of rye DArTs sequences were most similar to *Aegilops tauschii* mRNAs. Only one of them was matched to *Secale cereale* cds of resistant gene analog (putative disease resistance gene), and one to *Triticum aestivum* genomic sequence—also connected with resistance, in this case to fusarium head blight (FHB).

Sequence of DArT XrPt506905 from 3R showed similarity to predicted gene, namely, subtilisin-like protease. Subtilisin-like proteases (subtilases) are serine proteases and constitute the largest group of peptidases. Although several subtilases have been identified in plants (e.g., about 60 subtilase genes are known in *Oryza sativa* and *Arabidopsis thaliana*), most of their functions in plants remain unknown [23] (and bibliography therein). It is likely that subtilases contribute significantly to the developmental processes and signaling cascades in plants (Rautengarten et al. 2005, after [23]). For instance, the loss-of-function mutation in *ALE1* leads to abnormal leaf shape [24]. Marker XrPt506905, which is a predicted gene for subtilisin-like protein, in addition to the linkage with RL also showed a relationship with awn and grain length [21].

DArT XrPt507717, nearest to the peak of a QTL for RL from 3R, revealed similarity to protein-like COV2 mRNA. The role of COV2, inferred from the sequence or structural similarity to COV1 is stem vascular tissue pattern formation. COV1 is predicted to be an integral membrane protein that may be involved in the perception or transport of a signaling molecule that negatively regulates the differentiation of vascular tissue in the developing stem of *Arabidopsis* [25]. Marker XrPt507717, in addition to the linkage with RL also showed a relationship with awn length [21].

Two DArTs, XrPt401081 and XrPt398502 from 3R, has sequences homologous to jasmonate *O*-methyltransferase. This enzyme catalyzes the methylation of jasmonate into methyljasmonate, a plant volatile that acts as an important cellular regulator mediating diverse developmental processes and defense responses (http://www.uniprot.org/uniprot/Q9AR07). It is involved in the pathway of oxylipin biosynthesis, which is a part of lipid metabolism. To this end, 28 differentially expressed proteins related to rolled leaf traits were isolated and identified. Some of the proteins and genes detected are involved in lipid metabolism, which is related to the development of bulliform cells, such as phosphoinositide phospholipase C, Mgll, and At4g26790 [12].

Sequence of next DArT from 3R, XrPt507473, proved to be similar to *Fhb1*, a major FHB-resistant gene. *Fhb1* was fine mapped on the distal segment of chromosome 3BS of spring wheat (*Triticum aestivum* L.). One of the recent studies has reported that wheat *Fhb1* encodes a chimeric lectin with agglutinin domains and a pore-forming toxin-like domain conferring resistance to FHB [26]. Marker XrPt507473, in addition to the linkage with RL, also showed a relationship with awn length and stem thickness [21].

XrPt401454 from 5R showed homology to LRR receptor-like serine/threonine-protein kinase FEI11 mRNA. FEI1 is involved in the signaling pathway that regulates cell wall function, including cellulose biosynthesis, likely via an 1-aminocyclopropane-1-carboxylic acid (ACC)-mediated signal; a precursor of ethylene (http://www.uniprot.org/uniprot/C0LGF4). To date, 13 genes associated with rice RL have been isolated or cloned. The cytological mechanism of RL has been found to be largely related to the abnormal development of bulliform cells. NRL1 encodes cellulose synthase and plays a positive role in the regulation of bulliform cell development. In mutant rice plants that lack this gene, shrinkage is found in the area of the bulliform cells, thereby causing inward rolling of rice leaves [12].

Sequence of XrPt402531 from 5R demonstrated high similarity to predicted *A. tauschii* transcription factor bHLH79 (basic helix-loop-helix protein 79). The function of this factor is unknown; however, several transcription factors are known to be engaged in the establishment of abaxial/adaxial leaf polarity. For example, mutation in SLL1/RL9, a member of the KANADI family, encoding a transcription factor [27], leads to the failure of programmed cell death of abaxial mesophyll cells and the suppression of the differentiation of the abaxial cells, and finally to generate adaxially rolled leaves. ROC5 encodes a protein containing a leucine zipper domain, homologous to GLABRA2 in *Arabidopsis* which results in the negative regulation in the development of the bulliform cells. The number and size of the bulliform cells increased when ROC5 was knocked out, thereby leading to the generation of adaxially rolled leaves, whereas co-suppression of ROC5 resulted in abaxial RL [28].

Overexpression of a rice gene OsLBD3–7 that encodes a LBD family transcription factor promoted narrow and adaxially rolled leaves by decreasing the size and number of bulliform cells. OsLBD3–7 also upregulated the expression of negative regulators of bulliform cells, which implies that OsLBD3–7 acts as a suppressor of bulliform cell development [23].

The other rice gene ACL1 encodes an unknown protein with a conserved functional domain; OsZHD1 encodes a domain transcription factor with homologous zinc finger structure. These genes also play a positive role in the regulation of bulliform cell development, and overexpression of these two genes results in an increased number of bulliform cells, thereby causing outward rolling of rice leaves [12, 29].

XrPt402607 from 7R, marker nearest to the peak of QTL for RL and also marker linked with grain length and chlorophyll content [21], showed homology to predicted polyadenylate-binding protein-interacting protein 7-like gene. The poly(A) binding proteins (PABP) play an important role in the regulation of translation; however, the role of this particular factor is unknown.

Next marker from 7R, the peak of QTL for RL, DArT XrPt401480, seems to be homologous to the predicted gene encoding tubby-like F-box protein 12. Plants include a large number of tubby-like proteins (TLPs/TULPs). For example, there are 11 members of the tubby gene family in *Arabidopsis* [30], 14 in rice [31], 11 in poplar [32], 4 in wheat [33], and 8 in sorghum (http://www.ncbi.nlm.nih.gov). The existence of multiple TLPs implies their vital function in plants. F-box proteins regulate diverse cellular processes, including cell cycle transition, transcriptional regulation, and signal transduction. Lai et al. [30] have demonstrated that AtTLP9 interacts with ASK1 (*Arabidopsis* Skp1-like 1). According to them, F-box domain containing plant TLPs acting as transcription regulators should have cellular function activities of F-box proteins in signal transduction. AtTLP9 might participate in the abscisic acid signaling pathway [30]. The other example of F-box protein regulating plant growth and development include TIR1 acting in response to auxin [34].

The function of tubby-like F-box protein 12 encoded by genes of *Aegilops tauschii* and *Brachypodium distachyon*, homologous to XrPt401480, is unknown. However, some rice TLPs, especially OsTLP12, were probably involved in the abscisic acid and gibberellin signaling processes. This role might also be attributed to rye TLP12, because the same DArT was pointed as a marker linked to plant height [21].

XrPt390593 sequence from 7R was similar to vegetative cell wall protein gp1-like mRNA. The nature of cell wall proteins is as varied as the many functions of plant cell walls. Majority of the cell wall proteins are cross-linked into the cell wall and probably have structural functions. If this protein was associated with bulliform and/or hypodermis cells it might have an effect on RL, because these two types of cells are involved in RL in higher plants [1].

Although the roles described for the aforementioned markers as the potential genes that control RL are likely, their functions and association with RL and other traits should be verified in expression tests and will be studied during further research.

## Conclusion

The first localization of loci controlling leaf rolling (RL) on high density consensus genetic map of rye confirms the multigenic base of the trait stated for wheat and rice. Four stable quantitative QTLs on chromosomes 3R, 5R, and 7R were identified. Coinciding QTLs for RL and agronomic traits (e.g. drought tolerance) suggested pleiotropic effects of genes engaged in leaf rolling control. Four reproducible QTLs may be interesting for breeding purposes. The composite QTL analyze, the knowledge of the sequence of markers strongly linked to RL and the comparison of their homology with sequences data of related species allowed to indicate rye candidate genes controlling RL process.

## Additional files

Additional file 1: Matrix of coefficient correlation of leaf rolling (RL) in nine experimental variants, assessed in rye mapping population RIL-M. (XLSX 11 kb)

Additional file 2: Phenotypic data of rye mapping population RIL-M. (XLSX 66 kb)

## Abbreviations

AA: α-amylase activity
bHLH: Basic helix-loop-helix protein
DArT: Diversity array technology
GNPS: Grain number *per* spike
GNPS_DI: Grain number *per* spike drought index
GW: Grain weight per spike
HE: Heading earliness
MAS: Marker assisted selection
PH: Plant height
PHS: Pre-harvest sprouting
QTLs: Quantitative trait loci
RIL: Recombinant inbred line
RL: Leaf rolling
SCT: Spike compactness
SNPP_DI: Spike number per plant drought index
TGW: Thousand grain weight
TLP: Tubby-like proteins
